# Pixels as ROIs (PAR): A Less-Biased and Statistically Powerful Approach for Gleaning Functional Information from Image Stacks

**DOI:** 10.1371/journal.pone.0069047

**Published:** 2013-07-11

**Authors:** Jacob Pearson Keller, Kazuaki Homma, Peter Dallos

**Affiliations:** 1 Department of Communication Sciences and Disorders, Northwestern University, Evanston, Illinois, United States of America; 2 Department of Neurobiology, Northwestern University, Evanston, Illinois, United States of America; 3 Department of Otolaryngology, Head and Neck Surgery, Northwestern University, Chicago, Illinois, United States of America; Institute of Psychology, Chinese Academy of Sciences, China

## Abstract

Especially in the last decade or so, there have been dramatic advances in fluorescence-based imaging methods designed to measure a multitude of functions in living cells. Despite this, many of the methods used to analyze the resulting images are limited. Perhaps the most common mode of analysis is the choice of regions of interest (ROIs), followed by quantification of the signal contained therein in comparison with another “control” ROI. While this method has several advantages, such as flexibility and capitalization on the power of human visual recognition capabilities, it has the drawbacks of potential subjectivity and lack of precisely defined criteria for ROI selection. This can lead to analyses which are less precise or accurate than the data might allow for, and generally a regrettable loss of information. Herein, we explore the possibility of abandoning the use of conventional ROIs, and instead propose treating individual pixels as ROIs, such that all information can be extracted systematically with the various statistical cutoffs we discuss. As a test case for this approach, we monitored intracellular pH in cells transfected with the chloride/bicarbonate transporter slc26a3 using the ratiometric dye SNARF-5F under various conditions. We performed a parallel analysis using two different levels of stringency in conventional ROI analysis as well as the pixels-as-ROIs (PAR) approach, and found that pH differences between control and transfected cells were accentuated by ~50-100% by using the PAR approach. We therefore consider this approach worthy of adoption, especially in cases in which higher accuracy and precision are required.

## Introduction

Due to recent advances in the technology of fluorescent indicators, it is now possible to image a wide range of biochemical cellular changes with a variety of different delivery strategies (e.g., diffusion of small molecules, genetically-encoded proteins, etc.) and imaging modalities (simple intensity, ratiometric, FRET-based, etc.). Indicators are available not only for solutes such as calcium [1–4] and pH [5,6], but also for a number of other indicators of cellular function, such as cGMP [7] and hydrogen peroxide [8] and others. These methodological advances represent a giant step in our ability to query cellular function, since these experiments can be carried out relatively easily in living cells or tissues, varying conditions to determine the effects thereof. Therefore, through judicious use of these indicators, a multitude of heretofore-inaccessible questions can now be addressed quite readily.

One subset of these types of experiments that might have particularly profound ramifications is the characterization of individual proteins, especially those localized in the cell membrane, such as transporters and ion channels. From a pharmaceutical perspective, robotic assays of thousands of cell cultures can be carried out rapidly, enabling the screening of large libraries of compounds for possible future medicines. Further, once lead compounds are identified, binding affinities and kinetics can be similarly measured in living cells. But the relevance of these types of measurements is not limited to the development of pharmaceuticals: because essentially any fluorescence microscope can be used to perform these measurements, these techniques are used routinely to characterize protein function, even on the smaller scale. Analysis of these data traditionally involves identifying regions of interest (ROIs), and measuring changes therein over time or in response to a given experimental intervention. While this process does benefit from the visual recognition capabilities of the human observer, it can be subjective as well as cumbersome, especially in the high-throughput context. Furthermore, in the evaluation of the function of individual proteins, morphological cellular features used for ROI selection are largely irrelevant; what is more important for accurate and precise measurements are the statistical properties of the pixels included in the ROIs. This can be problematic, as many of the pixels included in an ROI can be statistically unreliable reporters of function, due to localization over moving cell edges, organelles, or nuclei, but these are nevertheless usually included in ROI-based analyses. What we propose herein is to consider each pixel a biochemical cuvette of sorts; from this perspective, the most important selection criteria are not those which indicate that the pixel is part of an apparent cell but those that establish statistical reliability. In this study, we investigate the efficacy of this new “pixels-as-ROIs” (PAR) paradigm in measuring transporter activity of a model protein, slc26a3, by comparing it with conventional ROI analysis. In so doing, we discuss the merits and drawbacks of various possibilities for statistical measures of pixel reliability. We find that this new approach provides a marked improvement in the measurement of slc26a3-mediated exchange activity, and this improvement should be generalizable to all similar cases.

## Methods

### Cell preparation and Data Acquisition

Opossum kidney (OK) cells (obtained from ATCC, line OK ATCC CRL-1840), chosen as the expression host because of their excellent adherence characteristics, were cultured in MEM and co-transfected with soluble cyan fluorescent protein (CFP) as a transfection marker and the human ortholog of the slc26a3/DRA protein (A3), a previously-characterized Cl^-^/HCO_3_^-^ exchanger [9,10], and plated onto poly-lysine coated glass coverslips. 24-48 hours after transfection, coverslips were secured in a 15 mm culture dish using vacuum grease to prevent slight motions. Solutions were perfused at room temperature onto the coverslips at 2 mL/min, and contained (mM): 125 NaCl (or 125 Na-gluconate), 5 K-gluconate, 1 MgCl_2_, 1 CaCl_2_, 25 NaHCO_3_, 20 HEPES equilibrated with 5% CO_2_ supplemented with 10 µM of the calcium ionophore calcimycin (Br-A23187) to confirm the effects of calcium on slc26a3 function described in [10]. Functional readout was achieved by using the ratiometric pH indicator dye SNARF-5F-AM (10 µM), incubated with cells for 20 minutes prior to image acquisition.

**Figure 1:**
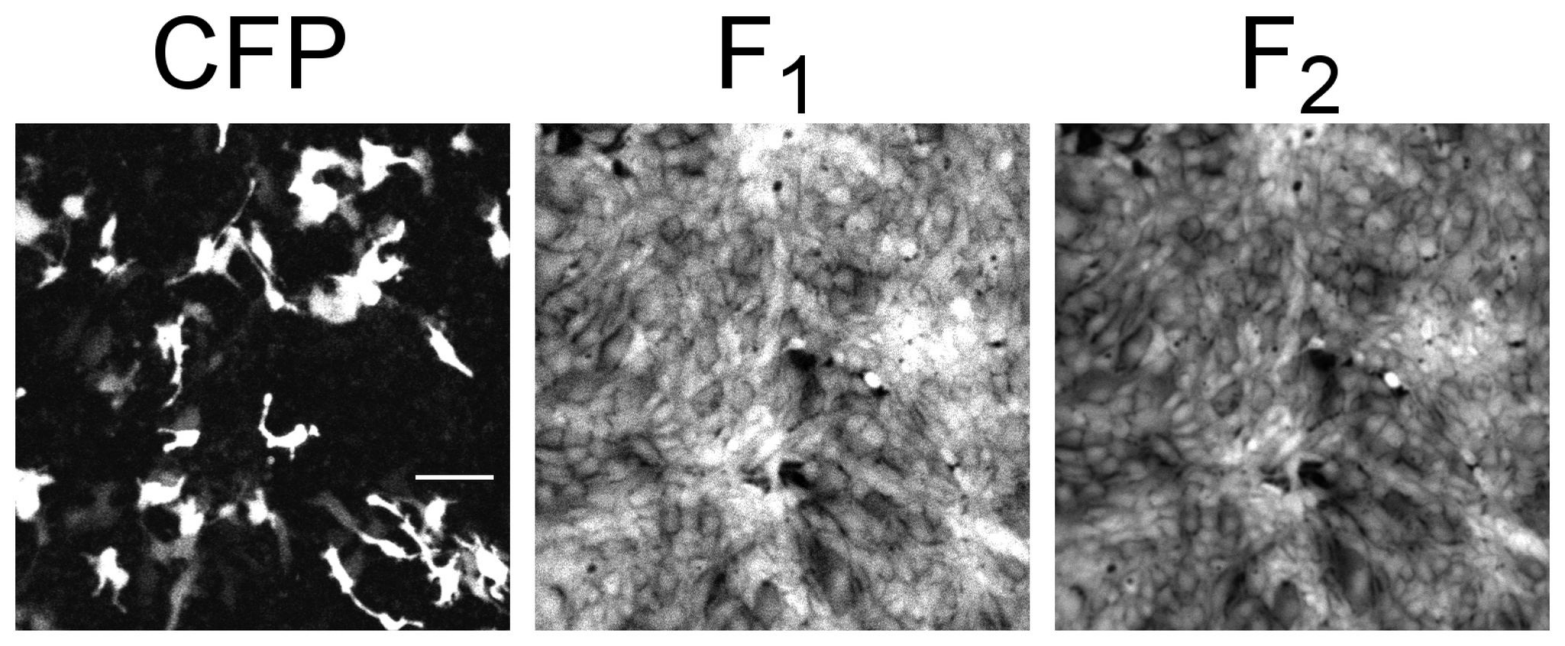
Sample images from dataset (raw data). Cultured opossum kidney (OK) cells were used in procedures described herein. CFP: representative fluorescence image of cells expressing cyan fluorescent protein, used herein as a slc26a3 transfection marker; excitation wavelength 457 nm and emission wavelength 495 nm long pass. White scale bar represents 100 µm, and is applicable to other two images, which encompass the same field of view. F1 and F2: representative fluorescence images at two wavelengths used for ratiometric monitoring of intracellular pH through the pH-sensitive intracellular dye SNARF-5F; excitation wavelength 561 nm and emission wavelengths 570-590 nm and 610-670 nm.

Images were acquired on a Nikon A1R laser resonant scanning confocal microscope every 10 seconds as three channels, one for measuring CFP fluorescence (excitation wavelength 457 nm and emission wavelength 495LP) and two for measuring the fluorescence ratios of the pH indicator dye (excitation wavelength 561 nm and emission wavelengths 570-590 nm and 610-670 nm). Resulting images were concatenated into a stack of 8-bit tif images using ImageJ [11] with a time-lapse series of images for each of three channels (Figure 1).

### Image Processing: Stack Alignment

**Figure 2:**
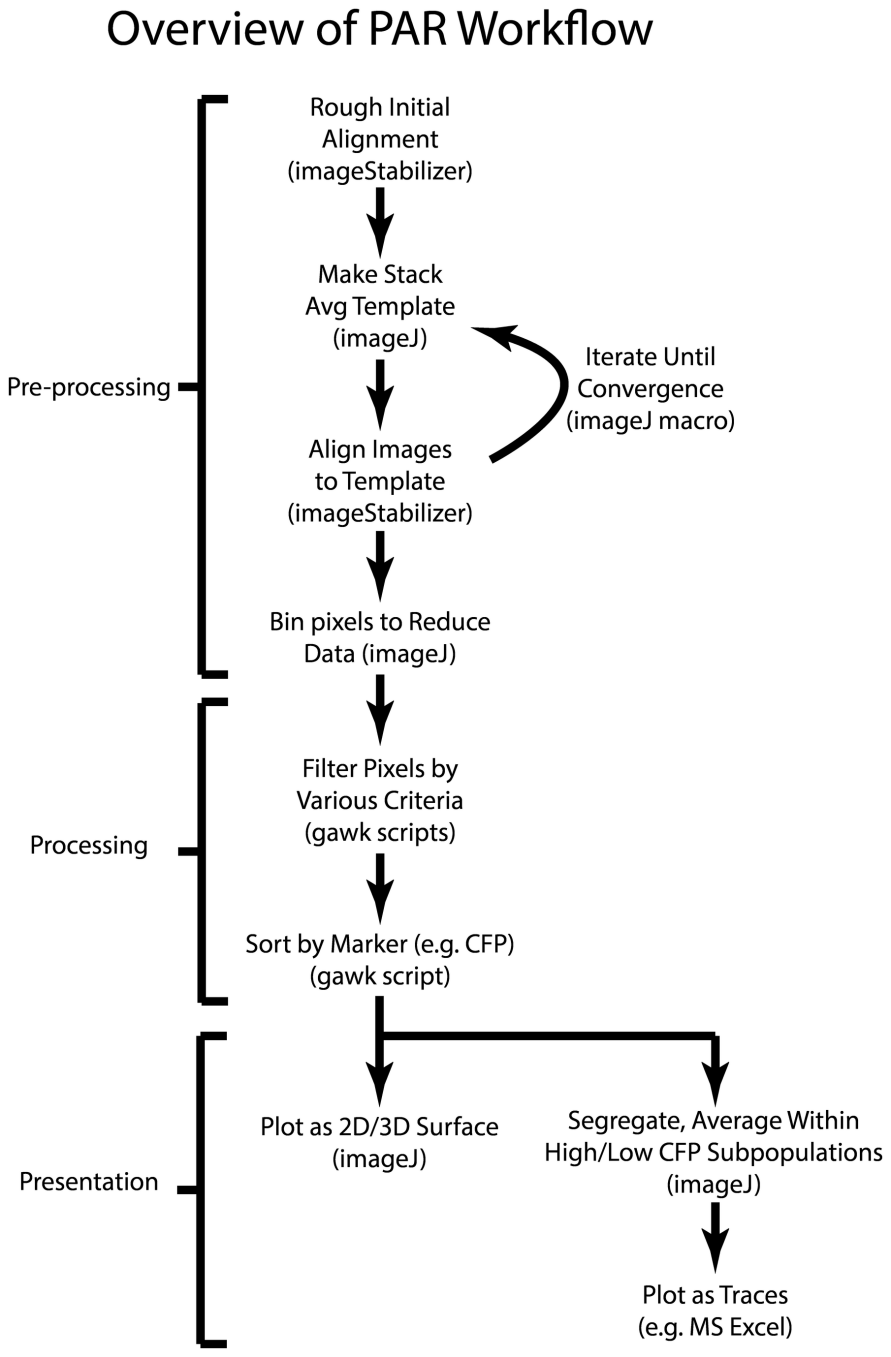
Schematic of workflow in PAR method. See text for detailed description.

The first stage in processing the images (see Figure 2 for schematic overview of the entire process) was alignment, and a good solution of the alignment problem was central for the success of the PAR method, which requires pixel location to be consistent over the course of the experiment. There were several types of obstacles in achieving good alignment. First, within each channel/wavelength, there was some degree of drift in the image frame over time, since experiments took 15-45 minutes. This was relatively easy to correct by using the imageJ plugin “image stabilizer” in the following iterative manner. First, a “stack-average” template image was created using the imageJ command “z-project,” and then each image in the stack was aligned to this template using image stabilizer. The stack was again averaged into a new template image, and then aligned to this new template. This process was iterated until convergence was achieved, as measured by the diminution of shifts during alignment between cycles to well below one pixel. For images with more drift than ours, it would presumably be helpful to begin either by aligning all images initially to the first image or perhaps an average of the first few frames, and then iterating as described. Since our images had relatively little drift, however, this was unnecessary and the alignment process was straightforward (but still certainly necessary).

As a potential obstacle to more general implementation of this algorithm, a problem at this stage might arise if cells change dramatically during the course of the experiment, leading to difficulties in alignment of the whole stack. Although this was not the case in our dataset, this possibility might be addressed by alignment of smaller, contiguous subsets of images first, then proceeding to a more global alignment. Whether the images need to be aligned globally at all, however, is a question that will be addressed below, as a variant of the PAR method (“framewise PAR”) sidesteps this need for continuity entirely.

The next issue in image alignment was aligning the three separate channels to each other. While the two SNARF-5F channels were very similar in appearance and therefore easy to register, the CFP channel was quite different, and had only a few features in common. This might be addressable generally at the experimental stage by adjusting the exposure parameters of the CFP channel to a level at which background fluorescence would be detectable, enabling visualization of image features which might be more similar to the other channels. Working, however, with the data we had already collected, our first resort was to try to use image stabilizer to align stack-average images of the independently-aligned SNARF and CFP channels. This failed, presumably due to a lack of similarity between the CFP and SNARF images, so we resorted to manual alignment to align what few stable “landmarks” we could find in the stack-averaged images. Once these were aligned, we could easily align each image stack to its mutually-aligned stack-average template, invoking the transitive property of alignment (i.e., if A is aligned to B, and B to C, A is aligned to C). Visual inspection of the alignment of the channels throughout the stacks cross-validated the efficacy of the procedure. While this aspect of processing presented some challenges, we were able to overcome them and assemble a precisely-aligned stack of images in three channels from which to derive our results.

### Image Processing: Binning

Our next decision was to what degree to “bin” the pixels: since the images contained >250,000 8-bit pixels each, and given processing limits currently imposed by various software and memory issues, we required at least 2x2 pixel binning to reduce the data to a manageable size. Although binning does introduce some blurring between the various classes of pixels (*viz*., blank pixels, untransfected-cell-containing pixels, and transfected-cell-containing pixels) it also affords some degree of stabilization of the data over time, especially in the inevitable case of imperfect image alignment at times, so we thought the process justifiable. It might be that with datasets that fluctuate more over time, a higher degree of binning would improve results, although we have not yet addressed this possibility in detail. In any case, after binning, we output the results to a table in which each column represented a single image, and each row a single (binned) pixel. In the case of our test dataset, this produced a table of ~65,000 rows and several hundred columns (~10^7^ data points). Although computing resources have dramatically increased over the years, enabling the very method being described, some limits still remain. Even these limits, however, are being quickly repealed.

### Image Processing: Pixel Rejection Criteria

The next stage, and perhaps the most critical, was the choice of appropriate statistical measures and cutoffs to filter out unreliable pixels whilst retaining the most informative pixels. Since the goal of this particular experiment was to determine what the effects of slc26a3 transporter expression were on pH changes, a critical requirement for these cutoffs was that they have equivalent effects on both high-transporter-expression (assumed to be equivalent to high-CFP-intensity) pixels and low- or no-expression pixels, so as not to introduce biases. As an obvious first criterion, we eliminated pixels in the SNARF-5F channels containing values exceeding the dynamic range of the camera at the settings used herein (“maxed-out”), as they were not reliable reporters of pH. This provided the set of pixels that might provide a valid functional readout.

**Figure 3:**
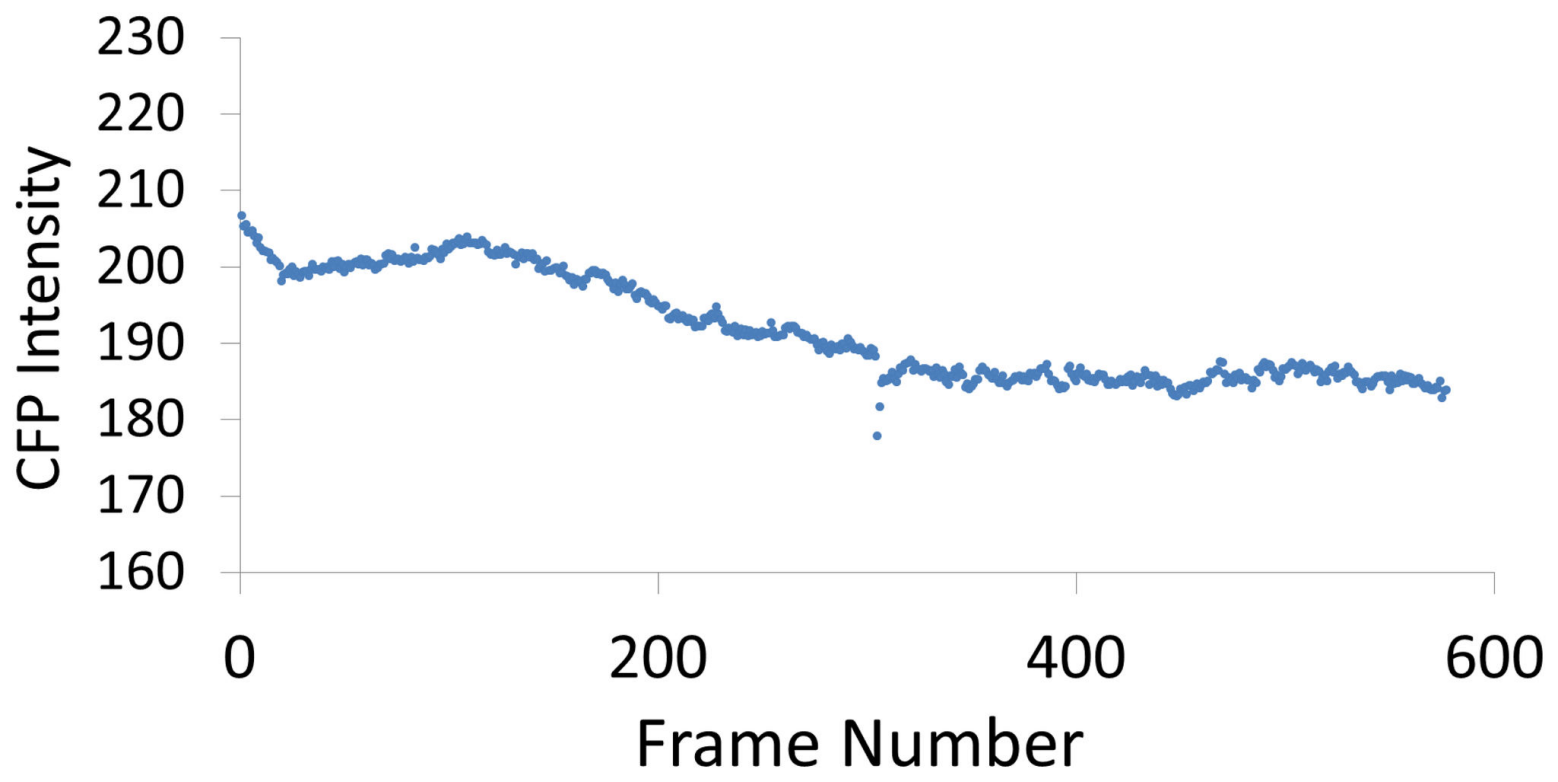
Decay of CFP intensity through the course of the experiment. For this plot, pixels were selected which never reached maximum values, yet whose average was above 100, to ensure monitoring of true CFP and not background signal. Note that the vertical axis begins at 160 and not 0, better to show the small changes (~10%).

Next, we used our *a priori* knowledge that for “well-behaved” pixels, the CFP channel should not change over the course of the experiment, beyond some presumably minor effects of photobleaching. Although this eventually became moot (see below), we sought as a precaution to quantify the magnitude of photobleaching effects by selecting only those pixels capable of measuring them, i.e., those which never reached maximum CFP intensity (255/255), and whose minimum never went below 100/255, ensuring that the pixel always contained some real CFP intensity. These criteria selected 1683/62492 (2.7%) pixels, which when averaged together and plotted, showed a moderate total decrement over the course of the experiment of ~10% of the original intensity (Figure 3). Perhaps of greater interest, however, was an unexpected, significant upwards-sloping phase of the intensity profile, occurring during a low-chloride phase of the experiment. Since slc26a3 is a chloride-bicarbonate exchanger, intracellular chloride would be expected to decrease during the low-chloride phase. We therefore tentatively interpreted this upturn as possibly a readout by CFP, whose fluorescence is probably at least weakly quenched by chloride [12], of the decreased intracellular chloride concentrations. Notably, this upturn did not occur in the calcimycin phase, consistent with inhibition of slc26a3-mediated chloride transport by calcium, and in agreement with the literature [10]. Thus, in addition to establishing that CFP was not drastically photobleached under the conditions of this experiment (~10%), we possibly discovered an unexpected measure that seemed to corroborate the activity of slc26a3 and its inhibition by intracellular calcium.

**Figure 4:**
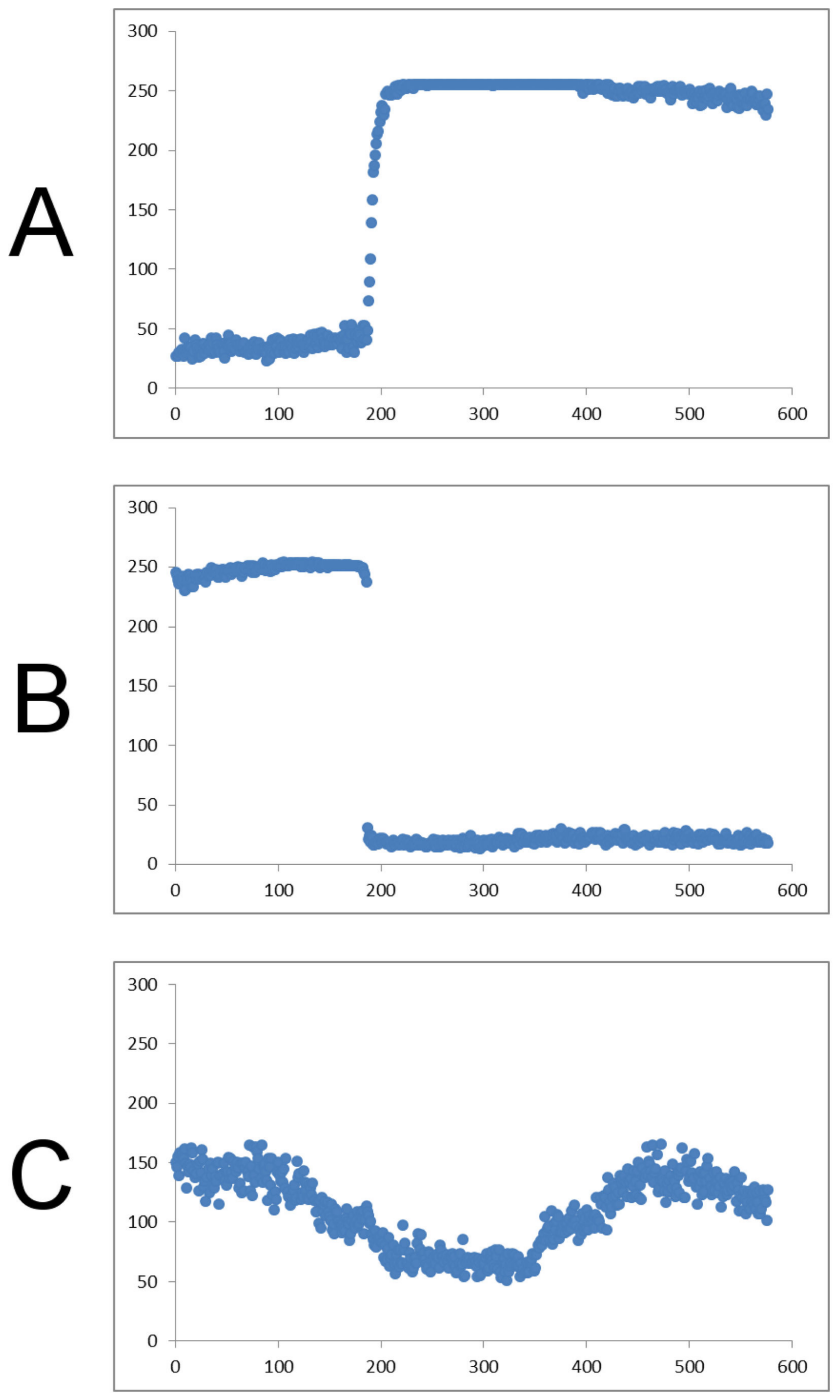
Traces of CFP intensities from three types of unreliable individual pixels, as a function of frame number. A and B: pixels which manifest dramatic changes in intensities, probably as a result of fluorescent material entering or leaving that pixel, both occurring approximately at frame 200. These pixels were detected quantitatively as outliers in maximum and minimum smoothed slopes, respectively. C: Generally noisy pixel, detected as an outlier in standard deviation of smoothed slope.

Assured of the stability of the CFP signal, we devised and evaluated several criteria for efficacy in selecting the most informative pixels. Upon inspection of CFP traces from individual pixels, we noticed in some cases features that seemed to indicate various types of pathological pixels, most notably pixels with large step-like discontinuities (Figure 4), probably caused by shifts in CFP-expressing cells into or out of the pixel under scrutiny. To eliminate these problematic pixels, we devised a measure for this phenomenon: the maximum magnitudes of slopes calculated for a sliding window of n time-points, with n = 50, a window size which worked well for distinguishing these discontinuities from simply noisy pixels. We also used the standard deviations of these slopes over the course of the experiment to eliminate pixels whose intensities wandered systematically in more than one direction, behavior inconsistent with pixels covering stable parts of the images. When we compared these measures, however, to the much-simpler measure of simple standard deviation, we found them to be highly correlated. Hence, we abandoned the possibly more sophisticated, powerful, and cumbersome measures in favor of more universal and easily-implementable ones. Using sigma cutoffs set empirically to eliminate most of the curve-discontinuities mentioned above resulted in two distinct sets of pixels at the extremes of possible intensity values, with most of the high-CFP pixels having values at- or near-maximal. Hence, as mentioned above, the question of photobleaching became moot: the high-CFP pixels were mostly “maxed-out,” (hence their low sigmas) and the lower-CFP pixels contained nothing susceptible to photobleaching. The use of a CFP-based cutoff, then, provided an excellent first-pass filter for establishing reliable pixels.

Another problem remained, however, which was the removal of pixels located on non-cell regions of the images. Whereas the high-CFP pixels were assuredly on cells, a measure was needed to ensure the same for the lower-CFP pixels lying on non-transfected cells. For this, we used the intensity values of the two SNARF-5F channels averaged across all time points in the experiment. High values would indicate of the presence of a cell in that pixel, as well as generally provide robust functional readout. While there was initially a small but significant difference between high-CFP and low-CFP pixels in the SNARF-5F mean intensities, we believe that this was due to a certain amount of non-cell-containing pixels in the low-CFP pixel population, which was not the case in the high-CFP pixel population. Since in our images, only approximately 10% of the area in the images appeared by eye to contain no cells, we set a cutoff that would eliminate 20% of the original pixels, ensuring a safe margin. While we could have used a still higher cutoff without much detriment, we felt that doing so would needlessly remove potentially useful information. The use, then, of the cutoffs described above yielded a set of pixels which were: 1) dependably located on cells, 2) highly-distinguishable regarding slc26a3 contents, and 3) robust and reliable reporters of pH. In a qualified sense, each of these pixels can be compared to a cuvette in a spectrophotometer measuring the transport characteristics of the protein in question, here slc26a3, or an appropriate negative control thereof. What is particularly remarkable, however, is that from one set of images corresponding to an experiment lasting approximately 30 minutes, we were able to derive data from thousands of “cuvettes,” whilst measuring even greater numbers (>10,000) of negative controls in the same preparation. Hence, from this exercise, one can perhaps glimpse the surprisingly immense information content of images.

## Results

### Slc26a3 Transporter Function: ROIs Vs. The PAR Approach

**Figure 5:**
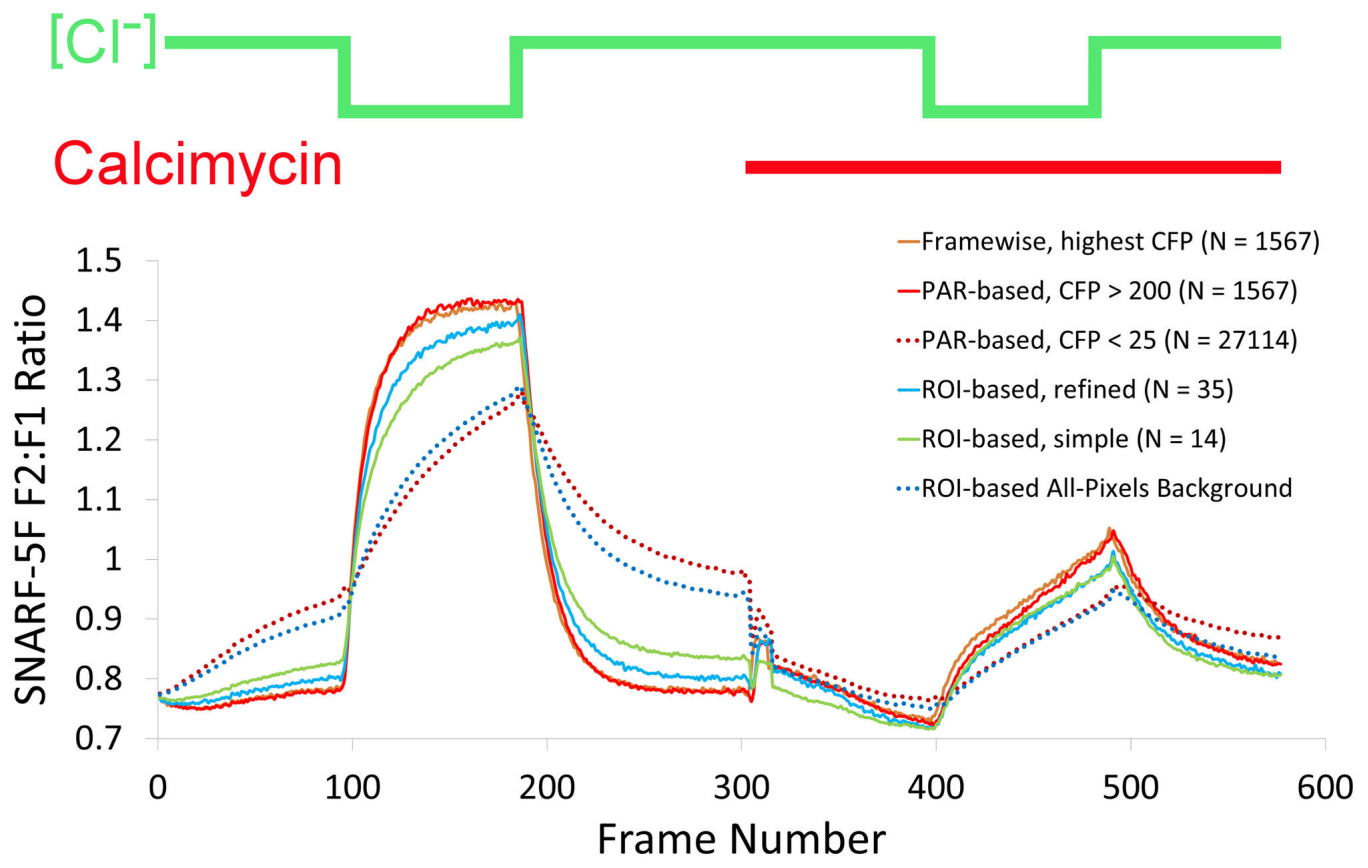
Comparison of results from various methods. Extracellular chloride concentrations are depicted above the traces as a green line, and the presence of the calcium ionophore calcimycin is indicated by a red bar. Dashed lines represent background/control measurements of untransfected cells from the same images, and solid lines represent measurements from slc26a3-containing cells.

**Figure 6:**
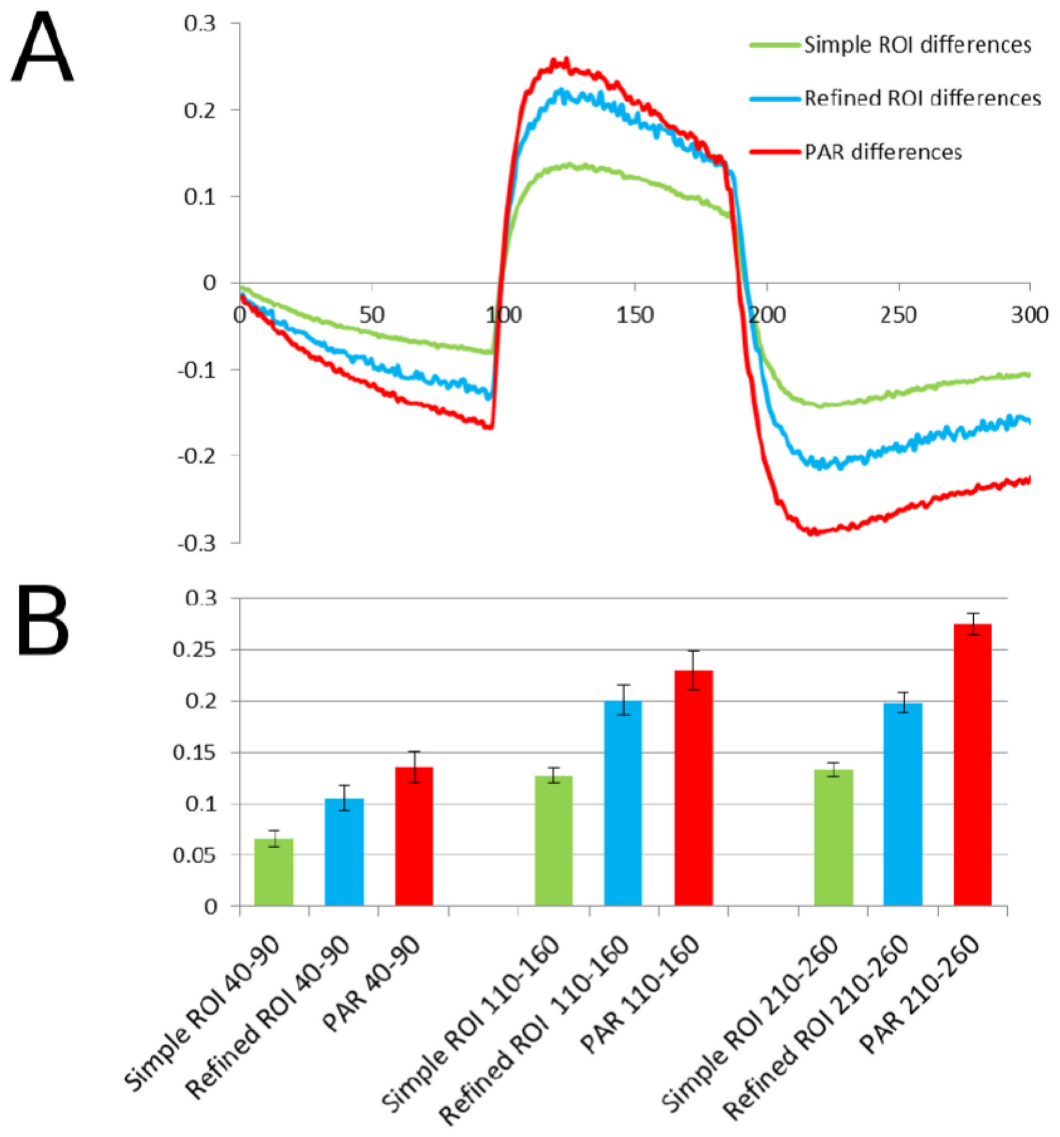
Statistical differences between methods. (A) Plots of differences between method-specific control and experimental curves shown in Figure 5. (B) Absolute values of differences in the 50-frame windows as indicated at bottom in labels. Error bars represent standard deviations derived from the values in the indicated 50-frame windows of the difference curves in (A).

Although there are many theoretical arguments in favor of using PAR instead of conventional ROIs, we wanted to determine whether in practice these advantages were significant. We therefore performed two ROI-based analyses on the same original images used for the pixel-based analysis described above: one very simple, and one with more refinements. In the simpler case (which we believe anecdotally to be common practice), we used images as taken directly from the experiment, without carrying out the iterative image alignment procedure described above. For selection of the ROIs, we used the first of the CFP images as a template, and set threshold levels by eye to what seemed appropriate for selecting transfected cells. We then selected the 14 largest and brightest ROIs, and measured them throughout the stack. For this part, since we were trying to re-create general practice, we deliberately did not try to establish a rigorous quantitative measure for selection, instead relying on our intuition. For a more refined processing method, we used the aligned but un-binned images from the pixel-based method, and also used the averaged CFP image stack as a template for ROI selection, using this time the explicit cutoff intensity of 200, and selecting all reasonable ROIs (35 in total) as delineated by this threshold to approximate more closely the pixel-based method. When the results of these two efforts were plotted together with those from the pixel-based method, initially-subtle but significant differences became apparent (Figure 5). First, while all methods showed a qualitative difference between transfected cells and background, the magnitudes of differences were greatest in the pixel-based method, followed by the refined ROI, and then the simple ROI methods. While in the case of the data herein, the inherent signal-to-noise seemed to be quite favorable, in less favorable cases, the choice of method might become more important for tracking subtler changes in function. In our case, the pixel-based method increased the maximum differences between the curves by 2-fold (re: simple ROI) or 1.5-fold (re: refined ROI) (quantified in three different frame-ranges in Figure 6), corresponding to Cohen’s d values (average of three frame-ranges in Figure 6) of 9.7 and 3.8, respectively, with size-of-effect R-values of 0.97 and 0.79. Another measure, paired two-tailed t-tests, yielded average p-values of ~10^-47^ (re: simple ROIs) and 10^-26^ (re: refined ROIs). It would appear that this high degree of significance is at least partly attributable to large sample sizes (50 time points), and that the significance would further increase were we to include individual pixel- and ROI-traces in the analysis, rather than averages thereof. These improvements are substantial, and might make a crucial difference in experiments exhibiting lower signal-to-noise ratios.

### Slc26a3 Transporter Function: Correction for Experimental Artifact

**Figure 7:**
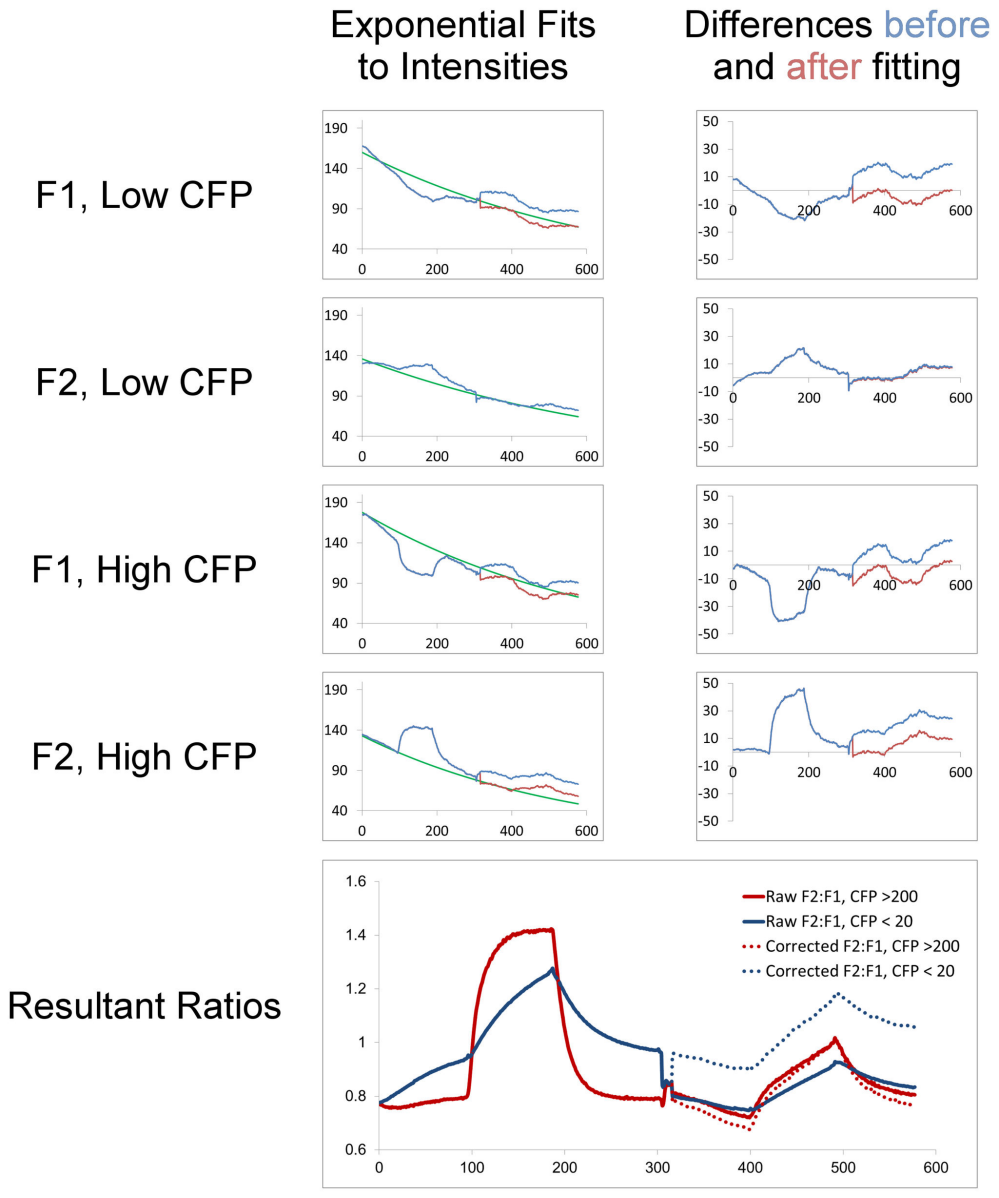
Correction procedure for raw fluorescence intensities. Left column of traces: raw (blue) and corrected (red) fluorescence intensities derived from pixel-groups indicated (F1 or F2, High- or Low-CFP), with exponential curves (green) fit to the high chloride phases in the pre-calcimycin stage of the experiment. Right column of traces: differences between exponential fits and observed intensities, before (blue) and after (red) the addition of a constant magnitude to minimize differences. Bottom: traces of F2: F1 ratios resulting from the raw (solid lines) or corrected (dashed lines) fluorescence intensities.

While the uncorrected data as presented in Figure 5 clearly demonstrate and corroborate the inhibitory effect of calcium described in [10], the observed discontinuity between the two stages (+/- calcimycin) suggested the possible introduction of a ratio-perturbing artifact somewhere in the transition. It seemed highly unlikely that such a sharp change in pH should occur in both cell cohorts (+/- A3 transfection), especially considering the gradual nature of the pH changes observed in all the other parts of the experiment. Specifically, at the middle of the traces, the baselines suddenly shift, and do not return to the levels seen before the introduction of calcimycin. While this might be a real effect brought about by the raising of intracellular calcium, it seemed possible that the discontinuity was in some way a measurement artifact. This led us to examine more closely the raw F1 and F2 fluorescence traces from the experiment (Figure 7, left column). Theoretically, for all data points across the image series having equal F2: F1 ratios (e.g., those in the equilibrated high-chloride phases), it would be expected to find uniform exponential decay in the fluorescence intensities, consistent with photobleaching effects. This was in fact observed in the initial, untreated phase of the experiment, as shown by the fit-ability to an exponential decay curve (a*exp(-b*x)) in the two high-chloride phases, which had roughly consistent ratios for extended periods (Figure 7). When we extended these fits into the calcimycin-treated phase, however, it became apparent that a single decay curve could not fit both the untreated and calcimycin-treated high-chloride phases. We first considered whether this might be due to real shifts in pH, but noted that for data points a given F2: F1 ratio, the sum of the F1 and F2 intensities should only decrease in the course of the experiment. This was clearly not the case when we examined our data, comparing the F1+F2 sums at data points having equal F2: F1 ratios. Apparently, then, something had happened to disturb the gradual decay in intensities in F1, F2, or both.

While we considered a large number of possible explanations for this discontinuity, we settled on the tentative hypothesis of a small amount of fluorescent material having been added concurrently with calcimycin addition—possibly the non-brominated isoform of calcimycin, which is fluorescent. The adoption of this hypothesis was in part driven by the observation that the deviation of the post-calcimycin raw intensities from the pre-calcimycin exponential fits was a roughly-constant amount. We therefore resorted to the relatively conservative strategy for its correction of subtracting a constant magnitude from the post-calcimycin raw intensities. This was carried out by first fitting exponential decay curves (Figure 7, green curves) to the two high-chloride segments in all four pre-calcimycin conditions (F1 and F2 for +/- transfected pixels). Then, a constant amount was subtracted from each of the post-treatment traces, such that the raw intensities from the high-chloride phases of each of them conformed most closely to the exponential decay curve derived from the pre-calcimycin part of the experiment (Figure 7, red traces). The traces of F2: F1 ratios resulting from this procedure were dramatically more continuous across the pre-post calcimycin transition (Figure 7 bottom, dashed vs. solid lines). As remarked above, while we feel that the data—even without correction—unequivocally support the conclusions of ref [10], we nevertheless feel that this procedure sharpened the accuracy of the measurements, bringing them closer to the true values.

## Discussion

### Slc26a3 Transporter Function: Physiological Explanation of Underlying Transport Processes

It may be wondered why in the conditions of this experiment the baselines differ between the transfected and untransfected cell populations (e.g., at frame ~90 in Figures 6, 7). To understand this, some background on slc26a3’s underlying physiology is required. Cells generally use intracellular carbonic anhydrases (CAs) to facilitate the interconversion of CO_2_ to HCO_3_^-^ and vice versa. The conversion takes place so readily that the intracellular CO_2_:HCO_3_^-^ ratio is strictly a function of intracellular pH according to the Henderson-Hasselbalch equation, with higher pH’s favoring lower CO_2_:HCO_3_^-^ ratios and vice versa. Since CO_2_ diffuses freely across cell membranes, its intracellular concentration will always be equal to the extracellular concentration. When extracellular CO_2_ concentrations rise, then, CO_2_ crosses the cell membrane and is converted by CAs into HCO_3_^-^ through addition of an OH^-^ ion, which results in transiently-lowered intracellular pH. In ordinary cells, this process reaches equilibrium fairly quickly, and cells rebound to neutral pH by intracellular buffering and other homeostatic mechanisms. This slow return to baseline can be seen in the untransfected cells during the initial phase of the experiment, i.e., frames 1-100 of Figures 6, 7.

**Figure 8:**
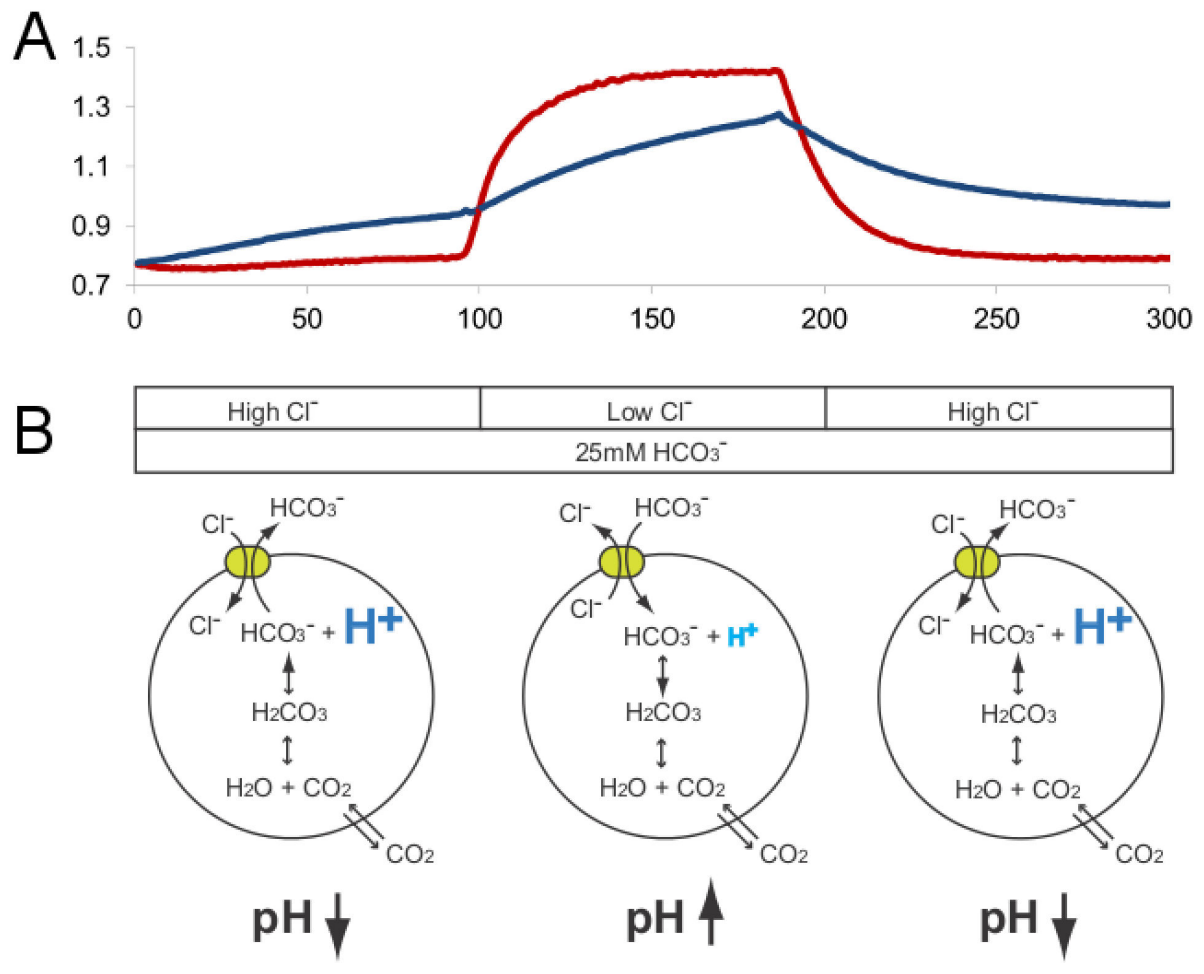
Physiological mechanisms underlying observed pH changes. Top: measured traces of F2: F1 intensity ratios in untransfected (blue) and slc26a3-expressing (red) cells, discussed herein. Bottom: diagrams of underlying bicarbonate-related physiological mechanisms described in text.

In the case of cells transfected with the HCO_3_^-^/Cl^-^ exchanger protein slc26a3, however, the situation is more complicated (Figure 8). Since slc26a3 exchanges HCO_3_^-^ for Cl^-^ passively, its activity depends on the concentration gradients of both ions. At the beginning of the experiment described herein, when the CO_2_/HCO_3_^-^ extracellular solution is introduced, the normal mechanisms of CO_2_/HCO_3_^-^ equilibration occur, leading all cells to have approximately equal initial intracellular pH values. At this phase, then, intracellular HCO_3_^-^ concentrations are all equal to the extracellular concentrations, assuming equivalent pH’s. There is, however, a substantial chloride gradient (~13:1 extracellular: intracellular) driving slc26a3 to export HCO_3_^-^. When HCO_3_^-^ is exported, the missing HCO_3_^-^ is immediately replenished by the conversion of free intracellular CO_2_ into HCO_3_^-^ at the expense of intracellular OH^-^, and this CO_2_ is then immediately replaced by free diffusion of CO_2_ into the cell across the cell membrane. Since intracellular OH^-^ is depleted in this process, intracellular pH decreases. This process reaches equilibrium when the intracellular pH is lowered to such a degree that very little free HCO_3_^-^ can exist, thus establishing a HCO_3_^-^ gradient in opposition to the Cl^-^ gradient, and halting further transport. Such is the situation when extracellular chloride levels are high.

When extracellular chloride levels are low, however, the situation changes. Now the chloride gradient is reversed, which drives HCO_3_^-^ into the cells. As this extra HCO_3_^-^ is converted into CO_2_ which then leaves the cells, it releases its OH^-^ group and elevates intracellular pH. Equilibrium is then reached only when intracellular pH is raised enough to accommodate levels of HCO_3_^-^ sufficiently high to counter the chloride gradient. Hence, in light of these considerations, the differences in baselines between the two types of pixels are exactly as expected, rather than anomalous.

### Frame-wise PAR

We also considered another novel approach, which was to relinquish the requirement of pixel continuity not only in space but also in time. In other words, given that each image of wavelength A was sufficiently aligned to its contemporaneous images at other wavelengths B and C, it is not necessary to retain the identities of pixels across the entire dataset. While surprising at first, this concept may have practical advantages. For example, in the case of substantial movements in the cells under scrutiny, the whole endeavor of functional measurement either by basic PAR or conventional ROIs might be jeopardized, since too few pixels would meet stability requirements across the entire experiment. With the framewise approach, however, the only requirement is that the imaged area be stable over the time-scale during which the images of various wavelengths are taken (a few seconds in the case of the data herein.) A corollary and drawback of this approach is that some of the statistical filtering described above is largely obviated (and impossible!), for all data points are now independent of each other, each consisting merely of three values for the intensity at each wavelength and a time stamp. We carried out this procedure on the same dataset as above, and compared the results to the other methods (Figure 5, orange trace). The performance was almost identical to the PAR method, which, as mentioned above, was better than conventional ROI analyses. In the case of these data, then, in which the cells remain stable over the course of the experiment, the benefits to this approach are insignificant, but in the many cases in which the cell population is more mobile, or over longer time courses, the framewise approach would certainly improve the measurements. Perhaps the only moderate drawback to this approach is the somewhat longer processing time required due to sorting framewise the millions of pixels involved.

## Conclusions/Summary

In the work presented herein, we have demonstrated the benefits of a new way of approaching at least a subset of image-based functional assays through abandoning, or at least transforming, the idea of ROIs. This approach has the potential advantages of having fewer preconceptions about the subject being imaged as well as higher statistical power due both to the potential use of higher numbers of cells (the need for seeing cell shapes is obviated) as well as the treatment of each pixel as an independent data point, although with the caveat that some pixels are obviously correlated with others through being part of a single entity (a cell or tissue). The concept of “number of measurements” or “N” in this context is thus bifurcated into the traditional “N” with reference to the number of cells and the “N” referring to the number of pixels, but confusion can be avoided simply by stating precisely what type of “N” is being reported. One potential weakness of the current form of the method is that it abandons some useful information, such as proximity of pixels in the original images or other morphological cues, but in exchange for this it receives a somewhat higher level of objectivity. In the future, the method would probably benefit by the re-incorporation of spatial and morphological information, which will be easy enough to implement through assignment to each pixel some sort of measure indicative of proximity, such as the intensities of surrounding pixels, to be used as a cutoff or weight in pixel selection. Although this type of information was not used in this paper, there were nevertheless significant improvements in the signal-to-noise ratio of the experiment compared to traditional ROI analyses: we found that in the case of our experiments on slc26a3, previously-reported inhibitory effects of elevations in intracellular calcium [10] could be detected more readily with less noise by using PAR. The current implementation of the PAR method would be least suited to cases in which morphological features or spatial patterns—especially rapidly-moving ones—are absolutely critical for identification of ROIs. For any dataset in which some mathematical feature of the constituent pixels can be used as a selection feature, however, the PAR method can be used. Additionally, even images with some degree of motion can be analyzed using the framewise PAR variant, provided they are relatively static over the time required to acquire images in the appropriate channels. An illustrative example might be the case of mitochondrial ratiometric pH measurements: whereas their motion over minutes would probably preclude using conventional PAR, framewise PAR would probably work well, considering that the acquisition of three channels of images (mitochondrial dye and two ratiometric pH wavelengths) takes at most a few seconds, during which time the mitochondria would be essentially stationary. It is our intention to push the limits of the method in these ways in future experiments. While in the case of the current experiment, the signal was large enough to be detectable by more conventional ROI analyses, under other circumstances, such as in the screening of expensive or rare compounds or where detection of small effects is required, the use of this method may be afford a means of doing what was heretofore impossible. Thus, in summary, the method proffers a substantial increase in signal-to-noise ratio for functional readout, allowing for more subtle functional changes to be detected, thereby opening new experimental possibilities.

## References

[1] Miyawaki A, Griesbeck O, Heim R, Tsien RY (1999) Dynamic and quantitative Ca2+ measurements using improved cameleons. Proc Natl Acad Sci U S A 96: 2135-2140. doi:10.1073/pnas.96.5.2135. PubMed: 10051607. 10051607 10.1073/pnas.96.5.2135 PMC26749

[2] Nagai T, Sawano A, Park ES, Miyawaki A (2001). Circularly permuted green fluorescent proteins engineered to sense Ca2+. Proc Natl Acad Sci U S A 98: 3197-3202. 11248055 10.1073/pnas.051636098 PMC30630

[3] Grynkiewicz G, Poenie M, Tsien RY (1985) A new generation of Ca2+ indicators with greatly improved fluorescence properties. J Biol Chem 260: 3440-3450. PubMed: 3838314. 3838314

[4] Akerboom J, Chen TW, Wardill TJ, Tian L, Marvin JS et al. (2012) Optimization of a GCaMP Calcium Indicator for Neural Activity Imaging. J Neurosci 32: 13819-13840. doi:10.1523/JNEUROSCI.2601-12.2012. PubMed: 23035093. 23035093 10.1523/JNEUROSCI.2601-12.2012 PMC3482105

[5] Buckler KJ, Vaughan-Jones RD (1990) Application of a new pH-sensitive fluoroprobe (carboxy-SNARF-1) for intracellular pH measurement in small, isolated cells. Pflugers Arch 417: 234-239. doi:10.1007/BF00370705. PubMed: 2084617. 2084617 10.1007/BF00370705

[6] Rink TJ, Tsien RY, Pozzan T (1982) Cytoplasmic pH and free Mg2+ in lymphocytes. J Cell Biol 95: 189-196. doi:10.1083/jcb.95.1.189. PubMed: 6815204. 6815204 10.1083/jcb.95.1.189 PMC2112339

[7] Niino Y, Hotta K, Oka K (2010) Blue fluorescent cGMP sensor for multiparameter fluorescence imaging. PLOS ONE 5: e9164. doi:10.1371/journal.pone.0009164. PubMed: 20161796. 20161796 10.1371/journal.pone.0009164 PMC2820094

[8] Belousov VV, Fradkov AF, Lukyanov KA, Staroverov DB, Shakhbazov KS et al. (2006) Genetically encoded fluorescent indicator for intracellular hydrogen peroxide. Nat Methods 3: 281-286. doi:10.1038/nmeth866. PubMed: 16554833. 16554833 10.1038/nmeth866

[9] Cheatham MA, Zheng J, Huynh KH, Du GG, Gao J et al. (2005) Cochlear function in mice with only one copy of the prestin gene. J Physiol 569: 229-241. doi:10.1113/jphysiol.2005.093518. PubMed: 16166160. 16166160 10.1113/jphysiol.2005.093518 PMC1464211

[10] Muallem D, Ashmore J (2006) An anion antiporter model of prestin, the outer hair cell motor protein. Biophys J 90: 4035-4045. doi:10.1529/biophysj.105.073254. PubMed: 16565043. 16565043 10.1529/biophysj.105.073254 PMC1459494

[11] Schneider CA, Rasband WS, Eliceiri KW (2012) NIH Image to ImageJ: 25 years of image analysis. Nat Methods 9: 671-675. doi:10.1038/nmeth.2089. PubMed: 22930834. 22930834 10.1038/nmeth.2089 PMC5554542

[12] Kuner T, Augustine GJ (2000) A genetically encoded ratiometric indicator for chloride: capturing chloride transients in cultured hippocampal neurons. Neuron 27: 447-459. doi:10.1016/S0896-6273(00)00056-8. PubMed: 11055428. 11055428 10.1016/s0896-6273(00)00056-8

