# Correction: Pixels as ROIs (PAR): A Less-Biased and Statistically Powerful Approach for Gleaning Functional Information from Image Stacks

**DOI:** 10.1371/annotation/435abed9-4628-4699-b020-faa83a1ef3b7

**Published:** 2013-07-30

**Authors:** Jacob Keller, Kazuaki Homma, Peter Dallos

The name of the first author was given incorrectly. The correct name is: Jacob Keller. 

The correct citation is: Keller J, Homma K, Dallos P (2013) Pixels as ROIs (PAR): A Less-Biased and Statistically Powerful Approach for Gleaning Functional Information from Image Stacks. PLoS ONE 8(7): e69047. doi:10.1371/journal.pone.0069047.

